# Development and validation of QuadraSynth ML: a novel classifier for sepsis prediction

**DOI:** 10.3389/fcimb.2026.1846826

**Published:** 2026-07-23

**Authors:** Xiaoxiao Shen, Yao Huang, Xinpeng Li, Zhuojie Li, Juan Du, Dalin Wen, Xiaodong Zhao, Zilong Li, Jian-Xin Jiang, An-Qiang Zhang

**Affiliations:** 1 College of Bioengineering, Chongqing University, Chongqing, China; 2 Department of Trauma Medical Center, Daping Hospital, State Key Laboratory of Trauma and Chemical Poisoning, Army Medical University, Chongqing, China; 3 School of Medicine, Chongqing University, Chongqing, China; 4 Department of Radiology, Chongqing University Cancer Hospital, Chongqing Key Laboratory for Intelligent Oncology in Breast Cancer (iCQBC), Chongqing, China; 5 Guizhou Medical University, Emergency Department of Guizhou Medical University, Guiyang, Guizhou, China; 6 Yuyao People’s Hospital, the Affiliated Hospital of Ningbo University of Medicine, Zhejiang, China

**Keywords:** biomarker, machine learning, prediction, RalA, sepsis, trauma

## Abstract

**Background:**

Sepsis, a common lethal complication of post-trauma, hinges on early detection and diagnosis for effective treatment. The involvement of RalA in regulating inflammatory diseases has been noted, yet its specific relationship with sepsis remains unclear. This study aimed to identify specific biomarkers for post-traumatic sepsis by developing a composite early-warning model incorporating various biomarkers, including RalA. Such a strategy is of significant clinical importance, enabling preemptive measures in managing traumatic sepsis, thereby enhancing treatment outcomes.

**Methods:**

Since July 2019, laboratory variables were collected from trauma patients within 24 hours after admission, for a total of 11 variables. Plasma RalA expression was measured via ELISA. Using random stratified sampling, 243 patients from Center A were divided into a training cohort (n=170) and an internal validation cohort (n=73) at a 7:3 ratio. Features related to traumatic sepsis were identified using Boruta, LASSO, and univariate logistic regression. Based on these selected common features, a predictive model for traumatic sepsis was developed employing six traditional machine learning classifiers and one proprietary classifier. An external validation cohort of 84 patients from Center B was used. Model performance was assessed by AUC, and diagnostic metrics such as specificity, sensitivity, and accuracy were calculated.

**Results:**

A total of 327 patients from two centers participated in this study. The QuadraSynth ML model developed in this research demonstrated favorable diagnostic performance in the primary cohort, with an AUC of 0.902. The AUC for the internal validation cohort was 0.836, and for the external validation cohort, it was 0.840.

**Conclusions:**

Admission RalA levels represent a promising candidate biomarker for early sepsis detection, but further prospective validation is required. The newly developed QuadraSynth ML model exhibits encouraging ability to identify sepsis early in patients with severe trauma.

## 1 Introduction

Sepsis, a lethal complication commonly occurring in the post-traumatic stage, significantly endangers patient health and has a high mortality rate (Liu et al., 2022; Martin-Loeches et al., 2024). Infection control and organ function support serve as the primary treatment approaches for sepsis; however, no specific drugs have currently been approved for its treatment (Moore et al., 2025). While positive microbial culture results are the gold standard for sepsis diagnosis, the time-consuming nature of this process may delay treatment (Stein et al., 2023). Consequently, the development of a novel method for the early prediction of post-traumatic sepsis is urgently needed.

The identification of biomarkers, which are simple and rapid, aids in the early detection and prognosis of post-traumatic sepsis in clinical settings. Classic biomarkers such as procalcitonin (PCT), C-reactive protein (CRP), white blood cell count (WBC), and lactate (LAC) contribute to the early detection of sepsis (Gomathi et al., 2022; Póvoa et al., 2023; Varga et al., 2024). However, their specificity and sensitivity in assessing sepsis are limited (Li et al., 2023a). Extensive research has confirmed that predictive models combining multiple biomarkers enhance the predictive accuracy of sepsis. Lu et al. developed and validated a new risk prediction score for sepsis in trauma patients by identifying seven indicators, including CRP, for the early prognosis of post-traumatic sepsis (Lu et al., 2019). Therefore, exploring specific early-warning biomarkers for post-traumatic sepsis and developing comprehensive models with multiple biomarkers has significant clinical implications for advancing the level of treatment and implementing preemptive measures in the management of trauma-induced sepsis.

Srinivasan and colleagues conducted a genome-wide association study on 351 children with sepsis and 406 without sepsis, identifying significant target-level SNPs in RALA (Srinivasan et al., 2017), suggesting a possible association relationship between the Ras-related protein Ral-A (RalA) and sepsis susceptibility. RalA, a member of the RAL branch of the RAS superfamily, is a small G protein with GTPase activity (Xia et al., 2024). In addition, previous work had shown that RalA is involved in inflammatory signaling, including regulation of the NLRP3 inflammasome pathway. Wang et al. reported that RalA/levornidazole inhibits the inflammatory response by blocking NLRP3/ASC/pro-caspase-1 complex assembly and reducing cleaved-caspase-1 and IL-1β/IL-18 secretion (Wang et al., 2020). Based on these findings, RalA could be a biologically relevant and clinically meaningful candidate biomarker for trauma-induced sepsis. However, the expression of RalA in trauma-induced sepsis remains unexplored, and its viability as a biomarker for sepsis requires further investigation.

In this study, routine clinical indicators within 24 hours after admission from trauma patients in different regions, Chongqing (Center A) and Zhejiang (Center B), were collected. Dimensionality reduction was initially performed using Boruta, the least absolute shrinkage and selection operator (LASSO), and univariate logistic regression methods. Subsequently, six machine learning models—logistic regression (LR), K-nearest neighbors (KNN), support vector machine with radial basis function (SVM-RBF), random forest (RF), linear discriminant analysis (LDA), and extreme gradient boosting (XGBoost)—along with the proprietary quadratic optimization for synthetic machine learning (QuadraSynth ML) model, were employed to predict the early risk of sepsis in trauma patients, undergoing both internal and external validation. The results indicate that the QuadraSynth ML model, which incorporates the levels of RalA, creatinine (Cr), and PCT, has greater potential for the early identification of patients with sepsis than does the Sequential Organ Failure Assessment (SOFA) score alone.

## 2 Methods

### 2.1 Study participants

For the collection of peripheral blood samples and clinical data from patients with severe trauma, the clinical records of such patients from the China Trauma Biobank, dating from July 2019 to the present, were selected. The inclusion criteria were as follows: (a) single-region AIS ≥ 3, or multi-region injury with ISS > 16; (b) patients aged between 16 and 70 years; (c) admitted to the hospital within ≤ 48 hours post-injury; and (d) survival time ≥ 7 days post-injury. The exclusion criteria were as follows: (a) patients who died within 48 hours after admission; (b) pregnancy or lactation; (c) severe underlying disease of vital organs; and (d) immunosuppression before injury (use of various immunosuppressive drugs). At the time of admission, none of the included patients had been diagnosed with sepsis. The outcome of interest was sepsis occurring after admission during the subsequent hospitalization. To ensure diagnostic accuracy, sepsis was strictly defined according to the Third International Consensus Definitions for Sepsis and Septic Shock (Sepsis-3). Specifically, sepsis was identified by a suspected or documented infection accompanied by an acute increase in the SOFA score of ≥ 2 points. To ensure consistency, cases were reviewed using standardized criteria by one independent clinician.

### 2.2 Variables adopted for analysis and imputation

The demographic information of the enrolled patients included age, gender, and underlying diseases, including hypertension and diabetes. The laboratory variables included complete blood counts, coagulation profiles, liver function tests, and kidney function tests. The complete blood count includes the WBC, lymphocyte (LYM), monocyte (MONO), and neutrophil (NEU) counts.

The time from injury to hospital admission and routine clinical and laboratory variables were recorded within 24 hours post-admission. In cases where multiple measurements were obtained within 24 hours, the worst measurement was used for analysis. Biomarker sampling for RalA was performed separately upon admission, as described below.

Variables with more than 30% missing values were excluded from the analysis. For the remaining variables, missing values were imputed in two steps. First, when a baseline value was missing but a repeated measurement was available on the following day after admission and before the diagnosis of sepsis, this value was used as an adjacent time point estimate of the early post admission status. This approach was used because some routine laboratory tests were not completed immediately at admission but were obtained shortly thereafter in clinical practice. To reduce the risk of temporal information leakage, measurements obtained after the diagnosis of sepsis were not used for imputation. For the remaining missing values, random forest imputation was applied (Hong and Lynn, 2020).

### 2.3 Detection of RalA

Blood samples were collected and processed as described in the literature (Fan et al., 2025). The whole blood was immediately collected into EDTA-coated tubes upon admission and stored at 4 °C. Human blood samples were centrifuged at 3000 rpm for 5 minutes at 4 °C within one hour of collection, followed by plasma separation for RalA detection. The Human RalA ELISA Kit (EH11656) was purchased from Fine Biotech, and the assay was conducted according to the manufacturer’s instructions.

This kit is applied to the quantitative detection of human RalA in plasma samples. The reported assay range was 0.156-10 ng/mL, with an analytical sensitivity of 0.094 ng/mL, and no obvious cross-reactivity with related analogues. Briefly, standards and plasma samples were added to anti-RalA antibody-precoated wells and incubated at 37 °C for 90 min. After washing, biotin-labeled antibody was added and incubated at 37 °C for 60 min, followed by HRP-streptavidin incubation at 37 °C for 30 min. TMB substrate was then added for color development, and the reaction was stopped with stop solution. Optical density was measured immediately at 450 nm using a microplate reader. RalA concentrations were calculated from a standard curve generated using serial standards ranging from 0.156 to10 ng/mL.

### 2.4 Feature selection

This study delineates a structured approach for developing a stable feature set for sepsis prediction by employing three distinct feature selection methods. Initially, the Boruta method was used to enhance the existing feature set. This method involves supplementing the dataset with an equivalent number of randomly shuffled shadow features and utilizing a random forest algorithm to assess their importance (Li et al., 2026).

Boruta was implemented using the Boruta package settings, with a maximum of 100 iterations, a significance threshold of P = 0.1 for feature confirmation, and multiple comparison adjustment enabled. Only features that demonstrated greater importance than the highest-ranked shadow features and were confirmed by Boruta were selected, forming candidate feature set 1.

Following this, LASSO logistic regression was used to identify predictors associated with sepsis while reducing model complexity. The LASSO model was fitted using a binomial distribution, and the penalty parameter λ was selected by 10-fold cross validation in the training cohort using the default package settings. The selected λ value was 0.114. Variables with nonzero coefficients at the selected λ value were retained to form candidate feature set 2.

Additionally, univariate logistic regression was employed to discern features significantly associated with sepsis. A two-sided P value of less than 0.05 was considered statistically significant, and variables meeting this threshold were retained to form candidate feature set 3. The final feature set was conservatively defined as the intersection of the three candidate feature sets to retain predictors consistently supported by random forest-based importance, penalized multivariable regression, and univariable association.

All feature selection procedures were performed exclusively in the training cohort to avoid information leakage from the internal or external validation cohorts. Boruta, LASSO, and univariate logistic regression were used as complementary screening methods rather than independent modeling steps. Only variables consistently selected by all three methods were retained for subsequent model development, resulting in a compact feature set. This conservative strategy was adopted to reduce dimensionality, limit the number of candidate predictors relative to the number of sepsis events, and improve model stability.

The stability of the selected features was further assessed across 100 resampling iterations in the training cohort. The final retained variables showed high selection frequencies, including approximately 100% for RalA, 100% for Cr, and 99% for PCT. Variables outside the intersection were not reintroduced into the final feature set, because the predefined feature-selection strategy was designed to retain variables consistently supported across the three screening methods and to reduce *post hoc* variable selection.

### 2.5 Development and assessment of models

Based on the selected features, single-feature models were constructed, and an RCP model combining RalA, Cr, and PCT was developed. To evaluate the effectiveness of the RCP model, the ISS, Glasgow Coma Scale (GCS), Acute Physiology and Chronic Health Evaluation II (APACHE II), and SOFA models were also established for comparative analysis.

In the training cohort, each model was developed using six conventional machine-learning classifiers, including LR, KNN, SVM-RBF, RF, LDA, and XGBoost. In addition, QuadraSynth ML was evaluated as an open-source weighted ensemble classifier rather than a proprietary or black-box model. It was designed to identify an optimal combination of conventional machine-learning classifiers for low-dimensional clinical prediction tasks. Additional details on the algorithmic design, implementation, and reproducibility of QuadraSynth ML are provided in the Supplementary Methods.

The algorithmic workflow of QuadraSynth ML was as follows. First, each conventional classifier was trained in the training cohort using the selected clinical or laboratory features. The predicted probabilities generated by these classifiers were then used as transformed meta-features. In this way, the original feature space was converted into a model-output probability space. Therefore, QuadraSynth ML did not directly replace the conventional classifiers; rather, it synthesized their probability outputs and determined the most informative weighted combination.

The core of QuadraSynth ML is a quadratic-programming-based weight optimization procedure. For each candidate combination of classifier outputs, non-negative ensemble weights were estimated under the constraint that the total weight equaled one. This constraint allowed the final ensemble score to be interpreted as a weighted aggregation of the selected classifier probabilities. During optimization, the algorithm searched for the classifier combination and corresponding weight distribution that improved the separation between outcome classes in the training cohort while controlling sample-level deviations. The candidate combination with the highest training performance was selected as the final QuadraSynth ML model. After the optimal weights were obtained, the weighted ensemble score was further converted into a calibrated probability using logistic calibration fitted only in the training cohort.

Hyperparameter tuning for the conventional classifiers was performed only in the training cohort using cross-validation and predefined search ranges. The optimal hyperparameters were fixed before evaluation in the internal and external validation cohorts. For QuadraSynth ML, model selection consisted of two training-only steps: selection of the candidate classifier combination and estimation of the corresponding ensemble weights. The final decision boundary was based on the calibrated probability threshold determined from the training cohort and then applied unchanged to the internal and external validation cohorts.

To reduce information leakage and overfitting, feature selection, base-classifier training, hyperparameter tuning, classifier-combination selection, ensemble-weight estimation, and probability calibration were all performed exclusively in the training cohort. The internal and external validation cohorts were used only for independent model evaluation. In the validation cohorts, the learned base classifiers, optimized weights, and calibration parameters were fixed and directly applied without further model refitting, weight re-estimation, threshold adjustment, or recalibration.

Model performance was assessed using receiver operating characteristic (ROC) curve analysis, area under the ROC curve (AUC), sensitivity, specificity, and accuracy. The 95% confidence intervals were estimated using 500 bootstrap resampling iterations. The QuadraSynth ML code was compiled and executed using MATLAB R2022a. The implementation mainly depended on MATLAB Optimization Toolbox for quadratic programming and Statistics and Machine Learning Toolbox for model fitting and performance evaluation. To improve reproducibility, the complete source code, model-training scripts, evaluation scripts, example input format, package dependencies, and random seeds have been deposited in a public GitHub repository. The random seed was fixed throughout data partitioning, model training, hyperparameter tuning, bootstrap resampling, and performance evaluation. The exact code version used in this study is available at https://github.com/YaoHuang1123/ML.

To interpret the RCP model at the individual and global levels, SHAP (SHapley Additive exPlanations) analysis was conducted. SHAP values were calculated using the Tree SHAP algorithm for the XGBoost-based RCP model, based on the game-theoretic Shapley value concept, by which each feature is assigned an importance value reflecting its contribution to the model prediction.

### 2.6 Statistical analysis

All the statistical analyses were conducted using R software (version 4.3.0) and Python (version 3.9.0). Continuous variables are reported as the mean values and standard deviations (SDs). The normality of distribution and homogeneity of variance for continuous variables were assessed using the Shapiro-Wilk test and Levene’s test, respectively. In cases where the data conformed to a normal distribution and variance homogeneity, t-tests were used for between-group comparisons. Otherwise, the Mann-Whitney U test was utilized to evaluate differences between sepsis patients and non-sepsis patients. Categorical variables are presented in terms of number and proportion, with chi-square tests applied to compare differences across groups. A total of 243 patients from Center A were randomly stratified and divided into a training cohort (*n* = 170) and an internal validation cohort (*n* = 73) at a 7:3 ratio. Additionally, 84 patients from Center B composed an independent external validation cohort.

To formally compare the discriminative performance of the RCP model with established clinical scores, pairwise AUC comparisons were performed using the DeLong test. The RCP model was primarily compared with SOFA and APACHE II scores in the internal and external validation cohorts. Pairwise AUC comparisons between the RCP model and individual predictors or clinical scores were performed using the DeLong test, with the RCP model used as the reference within each cohort. *P* < 0.05 was considered to indicate statistical significance, and all the statistical tests were two-sided.

## 3 Results

### 3.1 Baseline characteristics of patients

This study included a total of 327 trauma patients. The cohort from Center A was divided into an internal training set and an internal validation set. Specifically, 170 patients (70.0%) from Center A were allocated to the internal training dataset, and 73 patients (30.0%) were allocated to the internal validation dataset; 84 patients from Center B served as the external validation dataset. The baseline data of the patients are presented in **Supplementary Tables 1**–**S3**. The average ages for the internal training set, internal validation set, and external validation set were 45.7 years (SD 10.54), 42.4 years (SD 12.67), and 46.1 years (SD 13.29), respectively. The number of patients diagnosed with sepsis in the severe trauma category was 47 (27.6%) in the training cohort and 20 (27.3%) in the internal validation cohort, with 30 (35.7%) patients in the external validation cohort. The initial 24-hour SOFA scores were 2.41(SD 1.93), 2.45 (SD 2.14), and 3.11 (SD 3.02); the ISS scores were 23.55 (SD 8.19), 24.44 (SD 9.62), and 25.39 (SD 6.60); the GCS scores were 14.3 (SD 2.28), 14.1 (SD 2.52), and 12.68 (SD 3.97); and the APACHE II scores were 7.26 (SD 5.90), 7.55 (SD 6.18), and 12.39 (SD 6.85). Male patients were predominant, comprising 129 (75.9%), 59 (80.8%), and 64 (76.2%) patients in each cohort, respectively.

### 3.2 Feature extraction and selection

**Figure 1:**
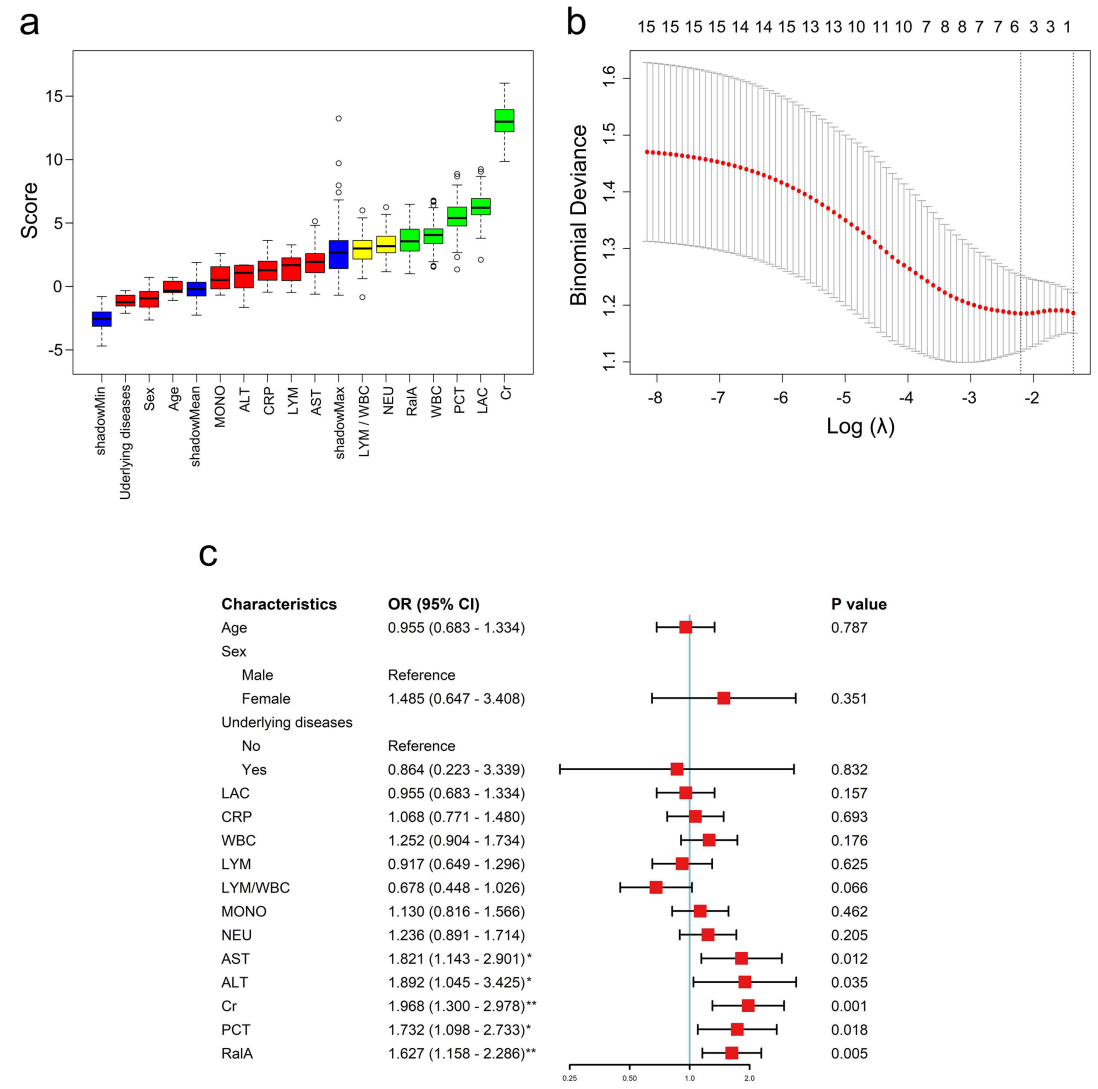
Extraction and screening of routine clinical features of post-trauma patients within 24 hours. **(a)** The Boruta method was utilized to select shadow features within the training cohort. **(b)** Binomial Deviance within the range of lambda. The binomial deviance was determined through cross-validation. The lambda parameter was chosen at its maximum value where the cross-validation error fell within a range of one standard error from the minimum. **(c)** Univariable logistic regression was employed to identify features significantly associated with sepsis.

The Boruta method identified RalA, WBC, PCT, LAC, and Cr as key predictive features for the identification of sepsis (**Figure 1a**). Similarly, the LASSO method identified RalA, PCT, LYM/WBC, Cr, and aspartate transaminase (AST) as significant features for sepsis identification (**Figure 1b**). Additionally, univariate logistic regression analysis indicated that the RalA, Cr, alanine aminotransferase (ALT), AST, and PCT were significantly correlated with the occurrence of sepsis (**Figure 1c**). By integrating the results from these three methods, RalA, Cr, and PCT were collectively recognized as the core predictive features for sepsis.

### 3.3 The performance of models

**Figure 2:**
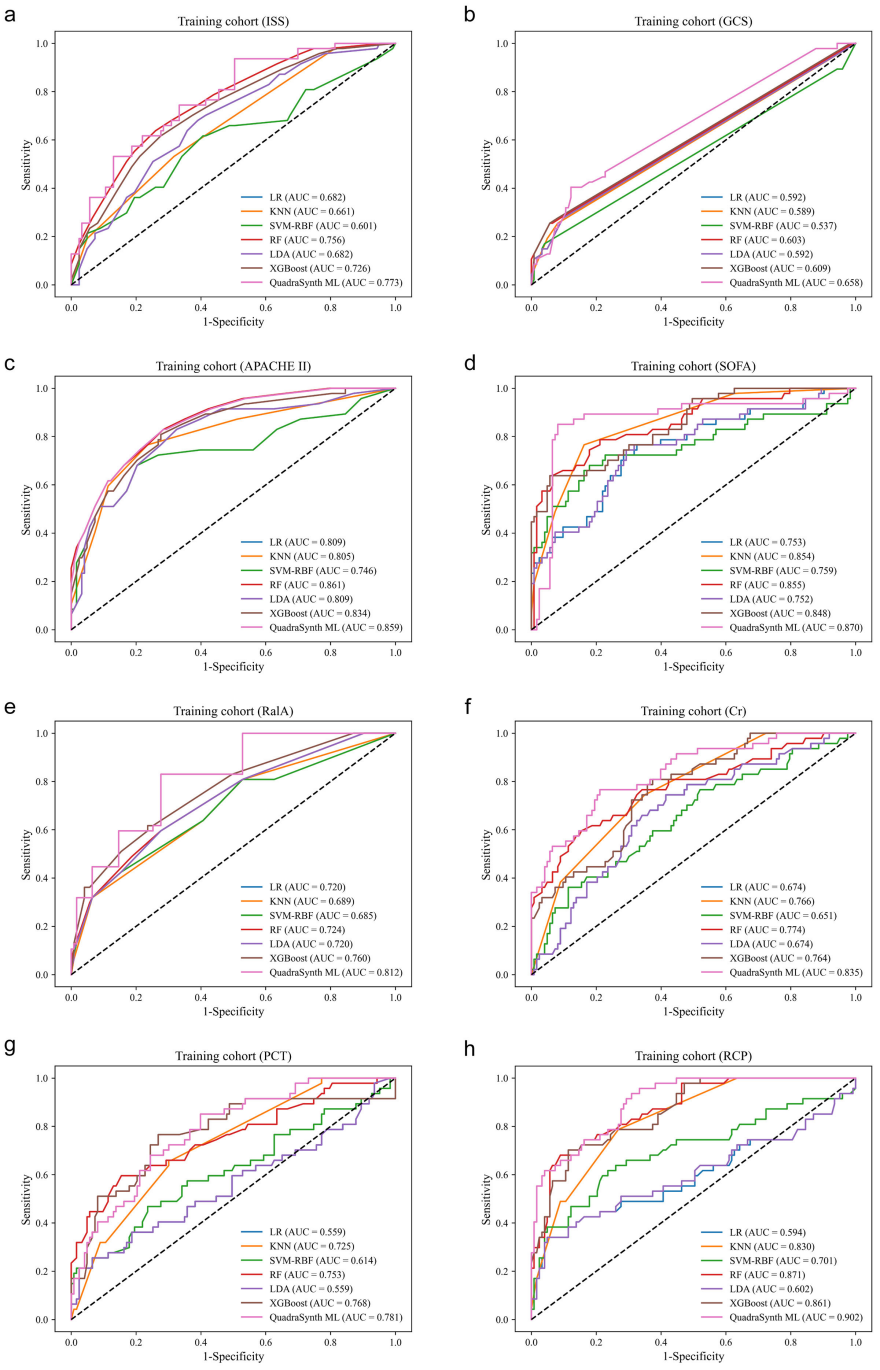
ROC curves of different machine-learning models for predicting the outcome in the training cohort. In each panel, the ROC curves of logistic regression (LR), k-nearest neighbors (KNN), support vector machine with radial basis function kernel (SVM-RBF), random forest (RF), linear discriminant analysis (LDA), extreme gradient boosting (XGBoost), and quadratic optimization for synthetic machine learning (QuadraSynth ML) model are displayed, with corresponding AUCs indicated in the legend. **(a)** ISS; **(b)** GCS; **(c)** APACHE II; **(d)** SOFA; **(e)** RalA; **(f)** Cr; **(g)** PCT; **(h)** RCP.

As shown in **Figure 2**, models developed using QuadraSynth ML showed favorable AUC values in the training cohort, ranging from 0.658 to 0.902. Compared with conventional classifiers, QuadraSynth ML generally yielded numerically higher AUC values across different prediction models. Therefore, QuadraSynth ML was used as the primary classifier for subsequent internal and external validation.

**Figure 3:**
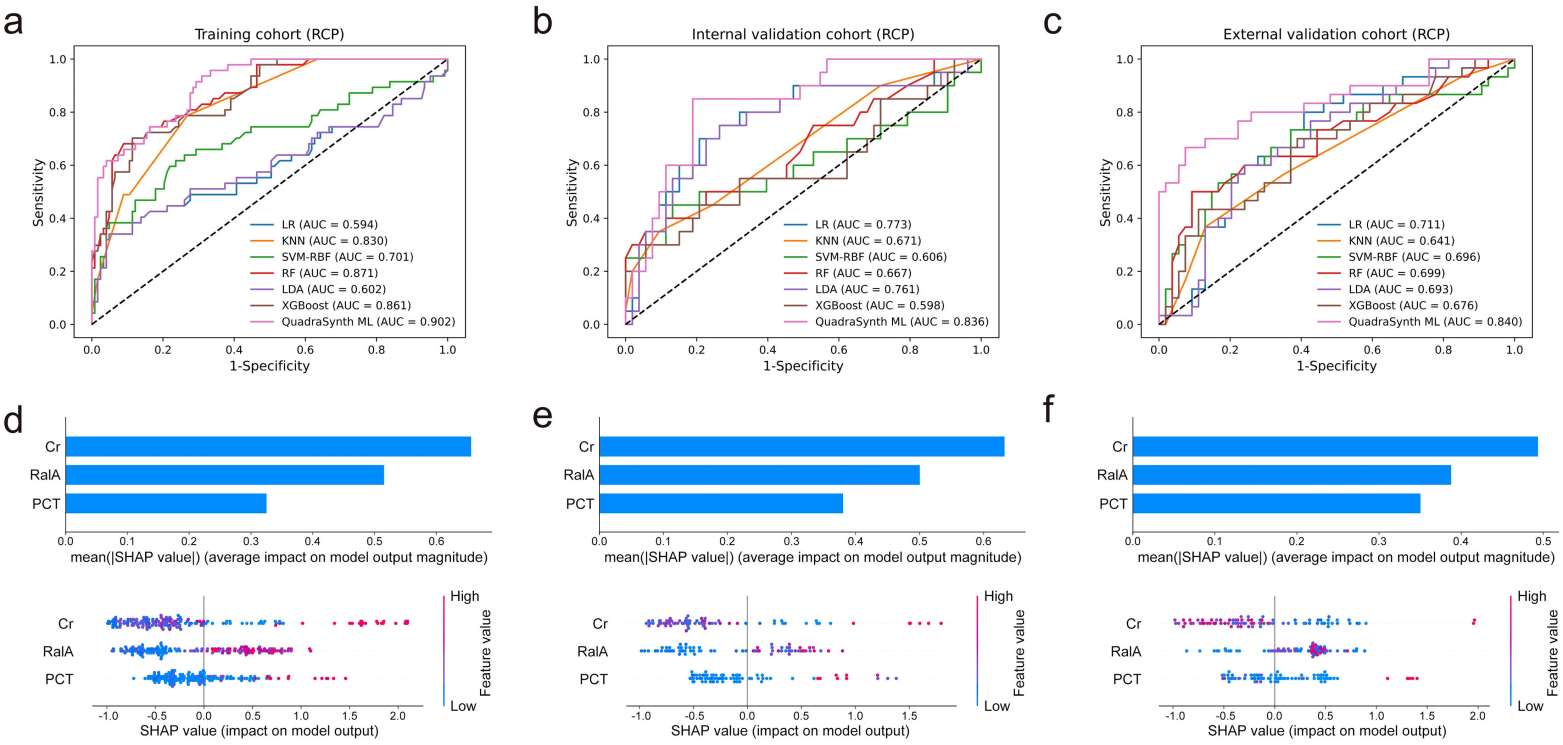
ROC curve analysis and SHAP analysis across the three cohorts of the RCP model. **(a–c)** ROC curve analysis across the three cohorts of the RCP model using six conventional machine learning classifiers. **(d–f)** Shap analysis across the three cohorts of the RCP model. Color indicates original feature values (blue = low, red = high). The x-axis shows SHAP values (impact on sepsis probability). Clinical units: RalA (ng/mL), Cr (μmol/L), PCT (ng/mL).

As indicated in **Table 1** and **Figures 3a–c**, the RCP model showed favorable predictive performance in both the internal and external validation cohorts, with AUC values of 0.836 and 0.840, respectively. Pairwise DeLong testing showed that the RCP model had significantly higher AUCs than ISS, GCS, RalA, and PCT in both validation cohorts, whereas the differences from SOFA and APACHE II were not statistically significant (**Supplementary Table 4**).

**Table 1:** The performance of the model.

Cohort	Model	AUC (95%CI)	Threshold*	Accuracy	Sensitivity	Specificity	PPV	NPV
Training cohort	ISS	0.773 (0.689, 0.840)	0.170	0.618	0.936	0.496	0.415	0.953
	GCS	0.658 (0.577, 0.742)	0.428	0.747	0.404	0.878	0.559	0.794
	SOFA	0.870 (0.792, 0.937)	0.276	0.900	0.851	0.919	0.800	0.942
	APACHE II	0.859 (0.795, 0.915)	0.259	0.747	0.830	0.715	0.527	0.917
	RalA	0.812 (0.743, 0.875)	0.223	0.753	0.830	0.724	0.534	0.918
	Cr	0.835 (0.759, 0.906)	0.247	0.782	0.766	0.789	0.581	0.898
	PCT	0.781 (0.695, 0.853)	0.193	0.671	0.851	0.602	0.449	0.914
	RCP	0.902 (0.853, 0.945)	0.134	0.759	0.936	0.691	0.537	0.966
Internal validation cohort	ISS	0.726 (0.594, 0.848)	/	0.699	0.850	0.642	0.472	0.919
	GCS	0.663 (0.557, 0.771)	/	0.781	0.400	0.925	0.667	0.803
	SOFA	0.807 (0.698, 0.908)	/	0.671	0.950	0.566	0.452	0.968
	APACHE II	0.783 (0.644, 0.913)	/	0.808	0.700	0.849	0.636	0.882
	RalA	0.754 (0.615, 0.872)	/	0.808	0.700	0.849	0.636	0.882
	Cr	0.788 (0.630, 0.915)	/	0.863	0.650	0.943	0.812	0.877
	PCT	0.706 (0.548, 0.863)	/	0.795	0.600	0.868	0.632	0.852
	RCP	0.836 (0.737, 0.930)	/	0.822	0.850	0.811	0.630	0.935
External validation cohort	ISS	0.724 (0.611, 0.832)	/	0.655	0.800	0.574	0.511	0.838
	GCS	0.664 (0.433, 0.780)	/	0.667	0.867	0.556	0.520	0.882
	SOFA	0.813 (0.704, 0.908)	/	0.821	0.667	0.907	0.800	0.831
	APACHE II	0.764 (0.641, 0.863)	/	0.738	0.800	0.704	0.600	0.864
	RalA	0.709 (0.594, 0.816)	/	0.714	0.667	0.741	0.588	0.800
	Cr	0.719 (0.593, 0.821)	/	0.762	0.467	0.926	0.778	0.758
	PCT	0.675 (0.416, 0.793)	/	0.774	0.467	0.944	0.824	0.761
	RCP	0.840 (0.739, 0.930)	/	0.833	0.667	0.926	0.833	0.833

AUC, area under the curve; CI, confidence interval; ISS, Injury Severity Score; GCS, Glasgow Coma Scale; SOFA, Sequential Organ Failure Assessment; APACHE II, Acute Physiology and Chronic Health Evaluation; RalA, Ras-related protein Ral-A; Cr, creatinine; PCT, procalcitonin; RCP, RalA, Cr, and PCT.

**Supplementary Figure 1** shows that the calibration curves of the prediction models were generally close to the ideal 45-degree reference line across the training, internal validation, and external validation cohorts, indicating good agreement between predicted and observed probabilities. The Hosmer-Lemeshow test results further suggested acceptable overall calibration for most models, although slight calibration deviations were observed for some models in certain cohorts. Moreover, SHAP analysis revealed the significance of three features within the RCP model (**Figures 3d–f**). A detailed visual analysis of the RCP model across the three cohorts indicated that higher values of RalA, Cr, and PCT increased the likelihood of classifying patients as having sepsis. Conversely, lower values of these indicators correspondingly elevated the probability of identifying patients as non-septic. This insight provides a deeper understanding of our RCP model and emphasizes the importance of specific physiological indicators in sepsis identification.

**Figure 4:**
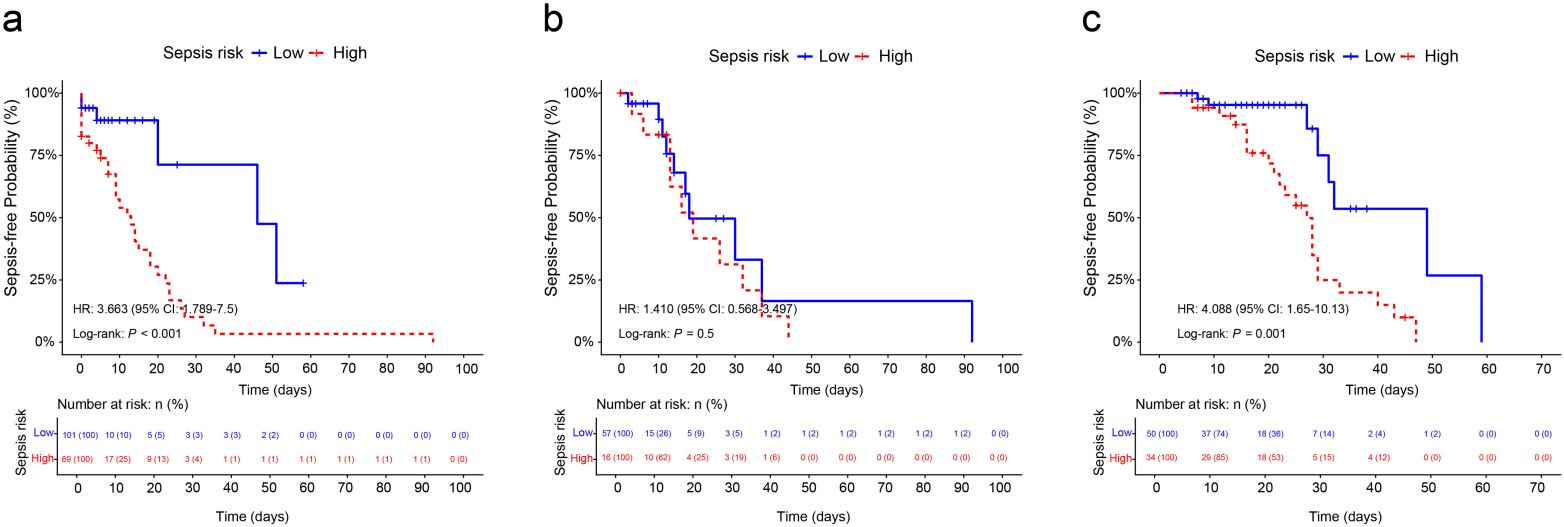
KM curve analysis across the three cohorts of the RCP model. **(a)** KM curve analysis of the training cohort (log-rank test, *P* < 0.001). **(b)** KM curve analysis in the internal validation cohort **(c)** KM curve analysis in the external validation cohort (log-rank test, *P* = 0.001).

Using the constructed RCP model, the probability values output for each patient serve as risk scores. The mean risk score of the training cohort (mean = 0.294) was used as the fixed cutoff value for categorizing patients into high sepsis risk and low sepsis risk groups. This cutoff was determined exclusively in the training cohort and applied unchanged to the internal and external validation cohorts. Kaplan-Meier curves revealed significant differences in the probability of sepsis between the two groups in both the training cohort and the external validation cohort (log-rank test, training cohort: *P* < 0.001; external validation cohort: *P* = 0.001; **Figures 4a, c**). In the training cohort, the high-risk group had a significantly higher risk of developing sepsis than the low-risk group (HR = 3.663, 95% CI: 1.789–7.5). Similarly, in the external validation cohort, the high-risk group showed a significantly increased risk of sepsis (HR = 4.088, 95% CI: 1.65–10.13). However, no significant difference was observed in the internal validation cohort (log-rank test, *P* = 0.5; HR = 1.410, 95% CI: 0.568–3.497; **Figure 4b**). Patients in the low-risk group exhibited a lower probability of developing sepsis.

## 4 Discussion

An increasing body of research indicates that RalA is involved in the regulation of tumors and inflammatory diseases. However, whether RalA can serve as an early warning biomarker for sepsis in trauma patients remains to be determined. In this study, the RCP model was developed using Boruta, LASSO, and univariate logistic regression methods to identify sepsis following trauma. In this cohort, the RCP model showed numerically higher AUCs than individual predictors and conventional clinical scores, suggesting its potential value for risk stratification. However, these findings require further validation.

Systemic inflammatory response syndrome (SIRS) or quick sequential organ failure assessment (qSOFA) scores are helpful clinical tools for early triage suspicion of sepsis. Yet, delays or misdiagnoses in sepsis diagnosis remain possible (Anand et al., 2019). There is an urgent need to explore new biomarkers for the early recognition and diagnosis of post-traumatic sepsis. Li et al. designed a wearable, battery-free wound dressing for sepsis patient monitoring using PCT as a biomarker (Li et al., 2023b). Mirko et al. used the neutrophil-to-lymphocyte ratio to predict mortality in elderly patients. At the same time, Liu et al. reported that an increased ratio on the day of admission was predictive of 28-day mortality in sepsis patients (Liu et al., 2021; Di Rosa et al., 2023). Research has shown elevated serum IL-36 levels in sepsis patients, possibly related to disease severity and mortality (Wang et al., 2023). Busse et al. reported that serum active renin levels are closely associated with mortality in critically ill sepsis patients, with higher levels indicating an increased risk of death (Busse et al., 2024). Pierre et al. identified monocyte distribution width as a novel biomarker for sepsis that is especially effective in assessing patients with a low pre-test probability of sepsis, improving diagnostic accuracy when combined with white blood cell count (Hausfater et al., 2021). These studies focused on classic biomarkers, inflammatory factors, and blood cells.

Zhou et al. chose the GBDT machine learning approach to develop a differential diagnosis model for AOSD and sepsis by combining clinical features and hematological indices (Zhou et al., 2023). Diao et al. used platelet-related genes to construct machine learning models, such as the LR, decision tree, and RF, to predict adverse outcomes in sepsis patients, evaluated and validated across six microarray platforms (Diao et al., 2023). With the development of artificial intelligence, machine learning has gradually been applied to screen and model clinical biomarkers. QuadraSynth ML was introduced in this study as an exploratory classifier for low dimensional clinical biomarker modeling. Its potential advantage may be related to its ability to capture limited nonlinear and interaction effects through a regularized quadratic decision structure, although the observed performance gain may also reflect differences in optimization and tuning strategies. Therefore, further studies using larger multicenter datasets, standardized nested cross validation, and prospective validation are needed to confirm its robustness and clinical applicability.

RalA is a small G protein with GTPase activity (Xia et al., 2024). It plays a crucial role in cellular biological functions, including signal transduction, gene expression, cytoskeletal dynamics, membrane transport, and cell division (Skorobogatko et al., 2018). RalA is involved in the development of various malignancies, such as liver, lung, ovarian, and squamous cancers (Moghadam et al., 2017; Gao et al., 2019; Konde et al., 2025). Zhao et al. reported that RalA GTPase regulates hematopoietic stem cell self-renewal and promotes chronic myelogenous leukemia, with overexpression in mouse bone marrow increasing the proportion of granulocyte-monocyte progenitors, hematopoietic stem cells, and multipotent progenitors (Yin et al., 2023). RalA also participates in tissue regeneration. Jonathan et al. identified Ral GTPases as critical regulators of mouse spinal cord myelin formation and maintenance (DeGeer et al., 2022). Neeraja et al. demonstrated the involvement of RalA in dendrite regeneration through transmembrane transport (Sanal et al., 2023). In addition, Zhu et al. reported that the RalGAPα1–RalA signaling pathway responds to stress-induced stimuli by enhancing the calcium reuptake capacity of the cardiac myocyte sarcoplasmic reticulum SERCA2a to maintain cardiac function, thereby protecting the heart by regulating calcium homeostasis (Zhu et al., 2022).

Severe trauma itself can induce a sterile systemic inflammatory response through the release of damage-associated molecular patterns (Vourc'h et al., 2018). Non-infectious inflammation may also lead to increases in CRP and PCT (Kumar et al., 2025; Xing et al., 2025), underscoring the difficulty of distinguishing infection-driven sepsis from trauma-induced sterile inflammation using inflammatory markers alone. Therefore, an important unresolved question is whether elevated RalA reflects infection-driven host dysregulation or a broader trauma-associated inflammatory response. RalA-related pathways may be involved in immune-metabolic regulation, inflammasome signaling, and endosomal trafficking. These processes are relevant to inflammation and immune responses. For example, EGFR has been shown to regulate Glut1 translocation to the surface of CD4^+^ T cells through the downstream TBK1/Exo84/RalA protein system, thereby inducing Warburg metabolism and promoting excessive CD4^+^ T-cell activation and apoptosis in sepsis (Huang et al., 2023). RalA has also been implicated in inflammatory signaling through regulation of NLRP3 inflammasome activation and IL-1β/IL-18 secretion, suggesting a potential link between RalA-related pathways and innate immune dysregulation (Wang et al., 2020). Another study reported that HuR-miRNA complexes bind to and stimulate endosomal RalA GTPase, facilitating the import of miRNAs into endosomes and their subsequent export in extracellular vesicles (Ghosh et al., 2024). In the present study, all samples used for RalA measurement were obtained from trauma patients. We specifically compared plasma RalA levels between trauma patients who subsequently developed sepsis and those who did not, to evaluate whether RalA could serve as a potential early warning biomarker for post-traumatic sepsis. Therefore, elevated RalA should be regarded as a candidate biomarker associated with post-traumatic sepsis, rather than a sepsis-specific marker. Future studies that include non-infectious controls, serial RalA measurements, and mechanistic experiments are required.

This study has several limitations. First, the retrospective design and relatively modest sample size may limit the generalizability of our findings and increase the risk of model overfitting. Although we used a compact three variable feature set and evaluated the model in both internal and external validation cohorts, its stability still requires confirmation in larger prospective studies. In particular, the current external validation should be regarded as a preliminary assessment of model transportability rather than definitive evidence of broad generalizability. Further multicenter external validation with larger and more heterogeneous patient populations is needed before the RCP model can be broadly applied in clinical practice. Second, QuadraSynth ML showed favorable numerical performance, but this study was not designed to definitively prove its superiority over established classifiers, and the observed advantage may partly reflect differences in optimization or tuning strategies. Third, although DeLong testing was added to formally compare AUCs, the differences between the RCP model and SOFA or APACHE II were not statistically significant in the validation cohorts. However, using only RalA, Cr, and PCT made the prediction process simple and convenient. In addition, decision curve analysis and net reclassification improvement were not performed in the current study, and the clinical net benefit of the RCP model should be further assessed in future studies. We acknowledge that calibration slope, intercept, and Brier score can provide complementary information, and these metrics will be considered in future studies with larger prospective cohorts. Finally, the intersection-based feature selection strategy was conservative and may have excluded potentially informative predictors, although it was adopted to reduce model complexity in the setting of limited sepsis events.

## 5 Conclusion

In conclusion, admission RalA is associated with subsequent sepsis risk in severe trauma patients and may represent a candidate biomarker for early risk stratification. The QuadraSynth ML model, incorporating RalA, Cr, and PCT, shows promising discriminatory performance for sepsis risk estimation in this retrospective cohort. However, these exploratory findings require multicenter prospective validation before clinical application.

## Data Availability

The original contributions presented in the study are included in the article/**Supplementary Material**. Further inquiries can be directed to the corresponding authors.
