# Supplementary material for: Development and validation of QuadraSynth ML: a novel classifier for sepsis prediction
Source: Front Cell Infect Microbiol. 2026 Jul 23;16:1846826. doi: 10.3389/fcimb.2026.1846826 (PMC13442006; doi:10.3389/fcimb.2026.1846826)
Supplement: Supplementary file 1 [file DataSheet1.docx]

**Supplementary Materials**

**Conceptual Architecture of QuadraSynth ML**

QuadraSynth ML was designed as a quadratic optimization-based classifier for low-dimensional clinical prediction tasks. In the present study, the input variables consisted of the three selected predictors, namely RalA, Cr, and PCT. Before model training, all input variables were standardized using parameters estimated from the training cohort. QuadraSynth ML then constructs a quadratic feature representation that includes the original predictors, second-order terms, and potential interaction terms among predictors. Based on this transformed feature space, the algorithm estimates a regularized decision boundary and outputs an individualized probability score for sepsis risk. This constrained structure was intended to capture limited nonlinear and interaction effects while avoiding the excessive flexibility of high-capacity models in a relatively small dataset.

QuadraSynth ML differs from conventional classifiers in several aspects. Compared with standard logistic regression, it allows a limited nonlinear decision boundary through quadratic terms. Compared with tree-based ensemble methods such as random forest and XGBoost, it does not rely on recursive partitioning, boosting, or a large number of weak learners. Compared with support vector machine models, its quadratic representation provides a more explicit feature-transformation structure for low-dimensional biomarker modeling. However, we acknowledge that the observed numerical advantage of QuadraSynth ML in the present study should not be interpreted as definitive evidence of algorithmic superiority. The performance gain may partly reflect differences in optimization strategy, hyperparameter tuning, and sample-specific characteristics. Therefore, QuadraSynth ML was regarded as an exploratory classifier in this study, and further validation using larger multicenter datasets, nested cross-validation, and prospective cohorts is needed to confirm its robustness and clinical applicability.

For n machine learning models, each model i outputs a prediction probability Pi(x) for a given sample x. The prediction probability of QuadraSynth ML is defined by Equation (1):

 Eq. (1)

Subsequently, the optimization problem is defined to maximize the AUC for the QuadraSynth ML model. This objective is articulated through the target function shown in Equation (2):

$Maximize\text{ AUC = }\int_{0}^{1} TPR\left(FPR\right)\text{ }d\left(FPR\right)$ Eq. (2)

TPR and FPR respectively represent the true positive rate and false positive rate. Subsequently, constraint conditions are defined, presented by Equation (3):

$\left\{\begin{matrix} \sum_{i=1}^{n} w_{i}=1 \\ 0\leq w_{i}\leq1 \end{matrix}\right.$ Eq. (3)

Finally, the aforementioned weight parameters are computed using the ‘quadprog’ function in MATLAB R2020a software. Detailed code for QuadraSynth ML can be found at https://github.com/YaoHuang1123/ML.

**Supplement Table 1. Baseline characteristics of trauma patients in the training cohort.**

| **Characteristics** | **All patients**  **(*n* = 170)** | **Trauma patients** | | ***P*** | **TEST** |
| --- | --- | --- | --- | --- | --- |
|  |  | **No (*n* = 123)** | **Yes (*n* = 47)** |  |  |
| Age，mean±SD，years（range） | 45.7±10.54 | 45.9±10.60 | 45.4±10.50 | 0.788561919 | T-test |
| LAC | 3.02±3.94 | 2.69±4.25 | 3.88±2.89 | 0.000122179 | Mann-Whitney |
| CRP | 49.62±55.14 | 48.59±55.45 | 52.32±54.84 | 0.524865267 | Mann-Whitney |
| PCT | 3.36±9.66 | 1.95±4.98 | 7.04±16.06 | 0.23341018 | Mann-Whitney |
| WBC | 13.63±5.55 | 13.28±5.46 | 14.57±5.73 | 0.09006974 | Mann-Whitney |
| LYM | 0.91±0.50 | 0.92±0.46 | 0.88±0.61 | 0.110904451 | Mann-Whitney |
| LYM/WBC | 0.07±0.05 | 0.08±0.05 | 0.06±0.05 | 0.017493796 | Mann-Whitney |
| MONO | 0.70±0.38 | 0.69±0.34 | 0.74±0.48 | 0.968036806 | Mann-Whitney |
| NEU | 11.62±5.19 | 11.31±5.17 | 12.44±5.22 | 0.124844428 | Mann-Whitney |
| AST | 133.10±216.86 | 99.09±111.12 | 222.11±358.95 | 0.003959156 | Mann-Whitney |
| ALT | 94.10±221.33 | 65.31±86.79 | 169.42±389.85 | 0.134086681 | Mann-Whitney |
| Cr | 78.45±35.54 | 72.20±20.52 | 94.81±56.08 | 0.024070378 | Mann-Whitney |
| APACHE2 | 7.26±5.90 | 5.49±4.43 | 11.91±6.74 | 4.44E-10 | Mann-Whitney |
| SOFA | 2.41±1.93 | 1.96±1.46 | 3.57±2.48 | 5.13E-06 | Mann-Whitney |
| GCS | 14.30±2.28 | 14.62±1.55 | 13.47±3.42 | 0.002614696 | Mann-Whitney |
| ISS | 23.55±8.19 | 21.64±7.24 | 28.05±8.71 | 0.000231174 | Mann-Whitney |
| RalA | 0.32±0.21 | 0.29±0.20 | 0.40±0.21 | 0.000459449 | Mann-Whitney |
| Number of days in the ICU | 4.85±11.59 | 1.93±6.40 | 12.49±17.40 | 4.14E-08 | Mann-Whitney |
| Prognosis |  |  |  | 0.0082174 | Mann-Whitney |
| No | 165 (97.1) | 122 (99.2) | 43 (91.5) |  |  |
| Yes | 5 (2.9) | 1 (0.8) | 4 (8.5) |  |  |
| Sex |  |  |  | 0.46192357 | Chi-square |
| male | 129 (75.9) | 91 (74.0) | 38 (80.9) |  |  |
| female | 41 (24.1) | 32 (26.0) | 9 (19.1) |  |  |
| Underlying diseases |  |  |  | > 0.99 | Chi-square |
| No | 158 (92.9) | 114 (92.7) | 44 (93.6) |  |  |
| Yes | 12 (7.1) | 9 (7.3) | 3 (6.4) |  |  |

LAC, lactate; CRP, C-reactive protein; PCT, procalcitonin; WBC, white blood cell; LYM, Lymphocytes; LYM/WBC, Lymphocyte/white blood cell; MONO, Monocytes; NEU, Neutrophils; AST, aspartate transaminase; ALT, alanine aminotransferase; Cr, creatinine; APACHE II, Acute Physiology and Chronic Health Evaluation; SOFA, sequential organ failure assessment; GCS, Glasgow coma scale; ISS, Injury Severity Score; RalA, Ras-related protein Ral-A.

**Supplement Table 2. Baseline characteristics of trauma patients in the internal validation cohort.**

| **Characteristics** | **All patients**  **(*n* = 73)** | **Trauma patients** | | ***P*** | **TEST** |
| --- | --- | --- | --- | --- | --- |
|  |  | **No (*n* = 53)** | **Yes (*n* = 20)** |  |  |
| Age，mean±SD，years（range） | 42.4±12.67 | 42.0±12.73 | 43.4±12.77 | 0.680802234 | T-test |
| LAC | 2.51±1.90 | 2.11±1.43 | 3.57±2.53 | 0.033357803 | Mann-Whitney |
| CRP | 45.69±39.92 | 43.05±38.41 | 52.66±43.96 | 0.457970638 | Mann-Whitney |
| PCT | 4.97±12.64 | 2.60±6.17 | 11.24±21.07 | 0.021411132 | Mann-Whitney |
| WBC | 13.17±5.96 | 13.34±6.25 | 12.74±5.22 | 0.8238179 | Mann-Whitney |
| LYM | 0.99±0.65 | 1.01±0.68 | 0.95±0.59 | 0.705947301 | Mann-Whitney |
| LYM/WBC | 0.09±0.06 | 0.08±0.05 | 0.09±0.08 | 0.916269948 | Mann-Whitney |
| MONO | 0.71±0.41 | 0.72±0.43 | 0.69±0.35 | 0.607681656 | Mann-Whitney |
| NEU | 11.23±5.44 | 11.38±5.71 | 10.82±4.75 | 0.945763324 | Mann-Whitney |
| AST | 167.20±538.67 | 86.18±72.06 | 381.93±1009.22 | 0.223105923 | Mann-Whitney |
| ALT | 88.34±158.98 | 63.43±57.61 | 154.35±283.51 | 0.322408076 | Mann-Whitney |
| Cr | 78.30±49.13 | 69.21±18.93 | 102.40±85.58 | 0.141044326 | Mann-Whitney |
| APACHE2 | 7.55±6.18 | 5.66±4.63 | 12.55±7.07 | 4.88E-05 | Mann-Whitney |
| SOFA | 2.45±2.14 | 1.68±1.22 | 4.50±2.69 | 1.09E-06 | Mann-Whitney |
| GCS | 14.10±2.52 | 14.42±2.00 | 13.25±3.48 | 0.087757938 | Mann-Whitney |
| ISS | 24.44±9.62 | 23.50±8.82 | 31.33±14.57 | 0.02141808 | Mann-Whitney |
| RalA | 0.42 ± 0.53 | 0.42 ± 0.57 | 0.43 ± 0.43 | 0.407260476 | Mann-Whitney |
| Number of days in the ICU | 8.40 ± 14.56 | 3.04 ± 6.21 | 22.60 ± 20.13 | 2.29E-09 | Mann-Whitney |
| Prognosis |  |  |  | 0.393675948 | Mann-Whitney |
| No | 71 (97.3) | 51 (96.2) | 20 (100.0) |  |  |
| Yes | 2 (2.7) | 2 (3.8) | 0 (0.0) |  |  |
| Sex |  |  |  | > 0.99 | Chi-square |
| male | 59 (80.8) | 43 (81.1) | 16 (80.0) |  |  |
| female | 14 (19.2) | 10 (18.9) | 4 (20.0) |  |  |
| Underlying diseases |  |  |  | 0.105422421 | Chi-square |
| No | 69 (94.5) | 52 (98.1) | 17 (85.0) |  |  |
| Yes | 4 (5.5) | 1 (1.9) | 3 (15.0) |  |  |

LAC, lactate; CRP, C-reactive protein; PCT, procalcitonin; WBC, white blood cell; LYM, Lymphocytes; LYM/WBC, Lymphocyte/white blood cell; MONO, Monocytes; NEU, Neutrophils; AST, aspartate transaminase; ALT, alanine aminotransferase; Cr, creatinine; APACHE II, Acute Physiology and Chronic Health Evaluation; SOFA, sequential organ failure assessment; GCS, Glasgow coma scale; ISS, Injury Severity Score; RalA, Ras-related protein Ral-A.

**Supplement Table 3. Baseline characteristics of trauma patients in the external validation cohort.**

| **Characteristics** | **All patients**  **(*n* = 84)** | **Trauma patients** | | ***P*** | **TEST** |
| --- | --- | --- | --- | --- | --- |
|  |  | **No (*n* = 54)** | **Yes (*n* = 30)** |  |  |
| Age，mean±SD，years（range） | 46.1±13.29 | 45.4±12.92 | 47.5±14.04 | 0.487965906 | T-test |
| LAC | 1.95±1.89 | 1.62±1.21 | 2.55±2.65 | 0.062022317 | Mann-Whitney |
| CRP | 19.79±31.79 | 19.96±33.77 | 19.49±28.42 | 0.655578308 | Mann-Whitney |
| PCT | 1.37±3.02 | 0.93±1.95 | 2.16±4.25 | 0.043726469 | Mann-Whitney |
| WBC | 15.53±6.68 | 15.20±6.99 | 16.12±6.18 | 0.306642509 | Mann-Whitney |
| LYM | 1.51±1.32 | 1.54±1.27 | 1.46±1.43 | 0.515443352 | Mann-Whitney |
| LYM/WBC | 0.11±0.11 | 0.12±0.11 | 0.10±0.09 | 0.333945039 | Mann-Whitney |
| MONO | 0.92±0.53 | 0.90±0.53 | 0.96±0.53 | 0.517594873 | Mann-Whitney |
| NEU | 13.03±6.38 | 12.67±6.72 | 13.67±5.78 | 0.302219282 | Mann-Whitney |
| AST | 97.94±134.23 | 92.70±123.43 | 107.37±153.57 | 0.304397463 | Mann-Whitney |
| ALT | 74.17±101.00 | 70.24±94.03 | 81.23±113.83 | 0.362624443 | Mann-Whitney |
| Cr | 60.92±18.64 | 60.64±16.21 | 61.43±22.65 | 0.740231081 | Mann-Whitney |
| APACHE2 | 12.39±6.85 | 10.54±5.46 | 15.73±7.87 | 0.001016147 | Mann-Whitney |
| SOFA | 3.11±3.02 | 2.24±2.55 | 4.67±3.21 | 0.000141768 | Mann-Whitney |
| GCS | 12.68±3.97 | 13.78±2.94 | 10.70±4.79 | 0.002892087 | Mann-Whitney |
| ISS | 25.39±6.60 | 23.78±5.71 | 28.30±7.19 | 0.004364492 | Mann-Whitney |
| RalA | 0.70 ± 0.51 | 0.71 ± 0.58 | 0.69 ± 0.37 | 0.367662477 | Mann-Whitney |
| Number of days in the ICU | 19.68 ± 11.46 | 16.33 ± 9.02 | 25.70 ± 13.00 | 0.000443304 | Mann-Whitney |
| Prognosis |  |  |  | 0.457556101 | Mann-Whitney |
| No | 78 (92.9) | 51 (94.4) | 27 (90.0) |  |  |
| Yes | 6 (7.1) | 3 (5.6) | 3 (10.0) |  |  |
| Sex |  |  |  | > 0.99 | Chi-square |
| male | 64 (76.2) | 41 (75.9) | 23 (76.7) |  |  |
| female | 20 (23.8) | 13 (24.1) | 7 (23.3) |  |  |
| Underlying diseases |  |  |  | > 0.99 | Chi-square |
| No | 65 (77.4) | 42 (77.8) | 23 (76.7) |  |  |
| Yes | 19 (22.6) | 12 (22.2) | 7 (23.3) |  |  |

LAC, lactate; CRP, C-reactive protein; PCT, procalcitonin; WBC, white blood cell; LYM, Lymphocytes; LYM/WBC, Lymphocyte/white blood cell; MONO, Monocytes; NEU, Neutrophils; AST, aspartate transaminase; ALT, alanine aminotransferase; Cr, creatinine; APACHE II, Acute Physiology and Chronic Health Evaluation; SOFA, sequential organ failure assessment; GCS, Glasgow coma scale; ISS, Injury Severity Score; RalA, Ras-related protein Ral-A.

**Supplementary Table 4. Pairwise AUC comparisons between the RCP model and other predictive models using the DeLong test**

| **Cohort** | **Model** | **AUC（95%CI）** | ***P* value** |
| --- | --- | --- | --- |
| Training cohort | ISS | 0.773 (0.689, 0.840) | 0.002 |
|  | GCS | 0.658 (0.577, 0.742) | < 0.001 |
|  | SOFA | 0.870 (0.792, 0.937) | 0.145 |
|  | APACHE II | 0.859 (0.795, 0.915) | 0.086 |
|  | RalA | 0.812 (0.743, 0.875) | 0.018 |
|  | Cr | 0.835 (0.759, 0.906) | 0.038 |
|  | PCT | 0.781 (0.695, 0.853) | 0.004 |
|  | RCP | 0.902 (0.853, 0.945) | Reference |
| Internal validation cohort | ISS | 0.726 (0.594, 0.848) | 0.015 |
|  | GCS | 0.663 (0.557, 0.771) | 0.003 |
|  | SOFA | 0.807 (0.698, 0.908) | 0.273 |
|  | APACHE II | 0.783 (0.644, 0.913) | 0.105 |
|  | RalA | 0.754 (0.615, 0.872) | 0.042 |
|  | Cr | 0.788 (0.630, 0.915) | 0.112 |
|  | PCT | 0.706 (0.548, 0.863) | 0.008 |
|  | RCP | 0.836 (0.737, 0.930) | Reference |
| External validation cohort | ISS | 0.724 (0.611, 0.832) | 0.012 |
|  | GCS | 0.664 (0.433, 0.780) | 0.002 |
|  | SOFA | 0.813 (0.704, 0.908) | 0.315 |
|  | APACHE II | 0.764 (0.641, 0.863) | 0.058 |
|  | RalA | 0.709 (0.594, 0.816) | 0.011 |
|  | Cr | 0.719 (0.593, 0.821) | 0.018 |
|  | PCT | 0.675 (0.416, 0.793) | 0.005 |
|  | RCP | 0.840 (0.739, 0.930) | Reference |
| Notes: AUC= area under the curve; CI=confidence interval; ISS, Injury Severity Score; GCS, Glasgow Coma Scale; SOFA, sequential organ failure assessment; APACHE II, Acute Physiology and Chronic Health Evaluation; RalA, Ras-related protein Ral-A; Cr, creatinine; PCT, procalcitonin; RCP=RalA, Cr, and PCT. | | | |

**Supplement Figure 1:** Calibration curves of the predictive models. The calibration curves depict the agreement between the predicted probabilities and the actual observed frequencies in the (a) training cohort, (b) internal validation cohort, and (c) external validation cohort. The 45-degree black dashed line represents the ideal perfect calibration. The Hosmer-Lemeshow (H-L) test was used to assess the goodness of fit, with a P value > 0.05 indicating good calibration.
